# Comparative diagnosis of bovine tuberculosis using single intradermal cervical tuberculin technique, conventional methods, enzyme-linked immunosorbent assay, and the gamma-interferon assay

**DOI:** 10.14202/vetworld.2022.1391-1397

**Published:** 2022-05-31

**Authors:** Sahar Hussein Abdalla Hekal, Amany N. Dapgh, Mai Badr-Eldien Abd-Elhafeez, Hassan Mohamed Sobhy, Fatma Ahmed Khalifa

**Affiliations:** 1 Department of Natural Resources, Faculty of African Postgraduate Studies, Cairo University, Giza, Egypt; 2 Department of Bacteriology, Animal Health Research Institute, Dokki, Giza, Egypt; 3 Central Administration of Veterinary Quarantine, General Organization for Veterinary Services, Dokki, Giza, Egypt; 4 Department of Infectious Disease - Animal Medicine, South Valley University, Qena, Egypt

**Keywords:** bovine tuberculosis, enzyme-linked immunosorbent assay, Gamma-interferon assay, *Mycobacterium tuberculosis*, single intradermal cervical tuberculin skin test

## Abstract

**Background and Aim:**

Bovine tuberculosis (TB) is a zoonotic disease that causes huge economic losses. This study aimed to compare the result obtained from the single intradermal test, conventional methods (culture and microscopy), gamma-interferon (IFN-γ) assay, and indirect enzyme-linked immunosorbent assay (ELISA) to diagnose bovine TB.

**Materials and Methods:**

This study evaluated 2913 animals from milk farms in Cairo, El-Sharkia, and El-Qalyubia Governorates by single intradermal cervical tuberculin technique (SICTT), ELISA, and IFN-γ assay.

**Results:**

Of the 2913 dairy cows surveyed, 3.7% yielded positive results. Culture prepared samples on Lowenstein-Jensen and Middlebrook 7H10 agar media yielded 52 (1.85%) isolates of *Mycobacterium* spp. from 2805 milk samples that yielded negative tuberculin reactions and 56 (51.85%) isolates of *Mycobacterium* spp. were recovered from 108 lymph node samples from positive cases. ELISA analysis of the sera of 108 positive SICTT reactors revealed that 94 (87.03%) and 97 (89.81%) animals were positive for bovine purified protein derivative (PPD-B) antigen and commercial polypeptide antigen, respectively. IFN-γ assays were performed on whole blood samples collected from positive SICTT reactors and showed that 103 (95.37%) animals were positive.

**Conclusion:**

*M. tuberculosis* complex may be isolated from raw milk and not all infected animals shed mycobacterial bacilli in their milk. The use of polypeptide antigen in ELISA provides better diagnostic efficacy than PPD-B antigen. The IFN-γ assay is more sensitive than both SICTT and ELISA. It should be used in parallel with SICTT to allow the detection of more positive animals before they become a source of infection to other animals and humans.

## Introduction

*Mycobacterium bovis* is the main causative agent of tuberculosis (TB) in cattle, but *Mycobacterium caprae* and *Mycobacterium tuberculosis* have also come to be regarded as causative agents [1]. A wide range of hosts can be infected by mycobacteria, complicating efforts at disease control [2]. TB, which is still considered a major health threat of public health concern, causes an estimated 8.6 million new cases and 1.3 million deaths annually [3]. Hence, the rapid and accurate diagnosis of TB is critical.

TB in cattle is a major zoonotic disease responsible for economic loss and negatively impacts the international trade of both live animals and their products. In humans, it causes approximately 3 million deaths every year. It affects domestic and wild animals and is characterized by progressive development of tubercles or characteristic granulomatous lesions in lung tissue, lymph nodes, or other organs [4]. Diagnosis of TB requires measuring either a humoral or cellular immune response. The tuberculin test is the test of choice globally and the most common test for bovine TB and international trade [5]. However, the tuberculin test is limited by variable specificity and sensitivity due to common skin reactive antigens that cause reactions in animals sensitized to non-tuberculous mycobacteria [6]. Others disadvantages of tuberculin test include the individual variation in test performance in injection, measurement ability, and test interpretation [7]. Alternative laboratory tests have been developed to overcome the drawbacks of the tuberculin test [8], including serological methods like the enzyme-linked immunosorbent assay (ELISA). *M. tuberculosis* is an intracellular pathogen, making it inaccessible to humoral antibodies. Hence, humoral immunity is less sensitive to bovine TB than cell-mediated immunity [9]. The gamma-interferon (IFN-γ) assay has been developed and used either alone or as an ancillary to the tuberculin test in TB diagnosis [10]. The IFN-γ assay has many advantages over the tuberculin test, such as early diagnosis of infected animals in endemic areas and easy standardization and interpretation [7].

This study aimed to compare results obtained from the single intradermal test, conventional methods (culture and microscopy), IFN-γ assay, and indirect ELISA for diagnosing bovine TB. The second aim was to rapidly detect animals positive for bovine TB to facilitate the prevalence assessment and control of risk factors associated with the occurrence and spread of TB through cow’s milk in Egypt.

## Materials and Methods

### Ethical approval

As per CPCSEA guidelines, a study involving clinical and postmortem samples does not require the approval of the Institute Animal Ethics Committee.

### Study period and location

The study was conducted in January and February 2021 in three Egyptian governorates (Cairo, El-Sharkia, and El-Qalyubia).

### Samples

Milk samples (n=2805) were collected from tuberculin-positive and tuberculin-negative cases. Lymph nodes were collected from tuberculin-positive animals. Serum samples were collected from tuberculin-positive reactors for ELISA serological testing. Whole blood samples were collected from tuberculin-positive reactors for testing by IFN-γ assay.

### Preparation of milk samples

Well-mixed milk samples (100 mL) were transferred to sterile tubes and centrifuged for 30 min at 1006 × g. The cream and milk serum were poured off, and the remaining sediment was examined for mycobacteria. This sediment was mixed thoroughly with an equal volume of 6% HCl and incubated at 37°C for 30 min. The mixture was recentrifuged for 30 min at 1006 × g. The supernatant was poured off, and the sediment was neutralized with 4% sterile NaOH using phenol red as an indicator (the change in color from pink to yellow indicates correct neutralization) [11].

Three to five drops of the decontaminated sediment were inoculated in two tubes containing Lowenstein-Jensen (L-J) medium (Biolife^®^, Italy) with and without sodium pyruvate and Middlebrook 7H10 agar (Difco^®^, USA). Inoculated McCartney tubes were sealed, labeled, and incubated at 37°C for at least 60 days with daily and then weekly observation (the Middlebrook 7H10 agar was incubated for 24 days). The type and rate of growth were recorded. Direct smears were made from isolated colonies, fixed by gentle heating, stained by the Ziehl-Neelsen method, and examined microscopically (Thermo Fischer, USA) for acid-fast organisms.

### Preparation of lymph node samples

Under aseptic conditions, lymph nodes with gross lesions were cut into small pieces, and fat was removed using sterile scissors in a sterile mortar containing sterile sand. The samples were crushed into a paste with sand. After adding 2 mL of sterile distilled water, the sample was ground into a suspension, and then 2 mL of 4% H_2_SO_4_ was added and incubated for 30 min. The sample was diluted with 16 mL sterile distilled water and centrifuged at 1006 × g for 20 min. The supernatant was decanted into 5% phenol. The sediment was used to make a direct smear, inoculated into a 4 mL L-J agar slant, and then incubated at 37°C for 3 weeks. Cultures were examined daily for 1 week and then weekly for 8 weeks [12].

A single intradermal cervical tuberculin skin test (SICTT) for dairy cows was performed according to OIE [13]. After clipping the hair in a narrow zone at the middle third of the neck, the injection area was marked, and skin thickness was measured with a certified caliper. The labeled site was inoculated with an intradermal injection of 0.1 mL bovine purified protein derivative (PPD-B). The reaction (swelling) was recorded 72±4 h post-injection by measuring differences in skin thickness (mm) [13].

The results were interpreted according to the Egyptian General Organization of Veterinary Services: Swelling <3 mm was considered negative, whereas an increase of 4 mm or more was positive. Reactions of 3-4 mm were considered indecisive and doubtful.

### Morphological identification of isolated mycobacteria:

Suspected colonies and direct smears of sediment were emulsified in a drop of 70% ethyl alcohol on a slide and spread to form a smear. The smear was allowed to air dry and then fixed by heat. The slide was then flooded using strong carbol fuchsin (Sigma-Aldrich, USA) and left on a heated slide holder for 5-7 min (steaming but not boiling). The slide was washed thoroughly with water and decolorized using acid alcohol for 1 min, washed with water, and flooded with counterstain (Loffler’s methylene blue) (BioWorld, USA) for 3 min. Finally, the slide was blot-dried and examined under an oil immersion lens to detect the shape, size, arrangement, and acid fastness.

### Serodiagnosis of bovine TB using ELISA

Reagents included PPD-B produced in the Veterinary Serum Vaccine Research Institute (Abbassia, Cairo, Egypt). Commercial polypeptide antigen (Prionics, AG Schlieren, Switzerland) was provided by the bovine TB unit of the bacteriology department, Animal Health Research Institute (Dokki, Giza, Egypt). Indirect ELISA was performed as described elsewhere [14].

The tested antigen was diluted (1:1000) in carbonate bicarbonate buffer at (pH 9.6), and 100 μL was added to the wells of a 96-well plate and then incubated overnight at 37°C . The plates were decanted, washed three times with ELISA wash (KPL) 20 × concentrate then blocked with 100 μL/well BSA (KPL) (1:10), incubated for 1 h at 37°C , and then washed three times with ELISA wash solution. Sample sera were diluted 1:20 in ELISA diluent (BSA 1:15), added (100 μL/well) to the coated plates, and then incubated at 37°C for 1 h. The microtiter plates were decanted and washed three times with ELISA wash. To each well, 100 μL of goat anti-bovine IgG-horseradish peroxidase conjugate (KPL) (Thermo Fischer, USA) (1:1000) was added, and the plates were incubated for 1 h at 37°C. The plates were washed three times with ELISA wash. ABTS substrate (100 μL/well) was added and incubated for 15 min. Results were read as optical density at 405 nm using a spectra III ELISA reader (Thermo Fischer).

### IFN-γ assay for diagnosis of bovine TB

The test was performed on whole blood samples. The test was standardized and verified according to the manufacturer’s instructions (Prionics Bovigam *M. bovis* Gamma Interferon Test Kit for cattle) (Life Technologies, Thermo Fischer).

#### Stage one: Whole blood culture

Blood samples (≥5 mL) were collected in heparin collection tubes from each animal. Aliquots (1.5 mL) of heparinized blood from each animal were dispensed into the wells of a 24-well tissue culture dish. Phosphate buffered saline (nil antigen control), avian PPD, or bovine PPD (100 μL/well) were added using an aseptic technique to the wells in triplicate. The plates were incubated for 16-24 h at 37°C in a humidified atmosphere. To harvest the samples, the culture dishes were centrifuged at 500 ×*g* for approximately 10 min. Approximately 500 μL plasma was carefully removed from above the sedimented red cells. Each sample was tested in duplicate.

### Stage two: Bovine IFN-γ EIA

Green diluent (50 μL) was added to the wells of a 24-well plate. Test and control samples (50 μL) were added. Control samples were added last. The plate was mixed by shaking for 1 min or pipetting up and down five times. The plate was covered with a lid and incubated at 25°C on a plate shaker at 600 rpm for 60 min. The plate was washed with wash buffer six times and then dried well. Freshly prepared conjugate reagent (100 μL) was added, and then the plate was covered with a lid and incubated at 25°C on a plate shaker at 600 rpm for 60 min. The plate was washed with wash buffer six times and then dried well. Freshly prepared enzyme solution (100 μL) was added, and then, the plate was mixed by shaking for 1 min or by pipetting up and down five times. The plate was covered with a lid and incubated at 25°C on a plate shaker at 40 × g for 60 min. Stop solution (50 μL) was added, being careful not to transfer chromogen from well to well and then mixed by gentle agitation. Absorbance was read within 5 min of terminating the reaction using a 450 nm filter with a 620-650 nm reference filter. Absorbance values were used to calculate the results.

## Results and Discussion

This study compared the diagnostic value of SICTT, conventional methods, ELISA, and the IFN-γ assay in bovine TB.

TB prevalence in three Egyptian Governorates (Cairo, El-Sharkia, and El-Qalyubia) is shown in Table-1. We found 108/2913 (3.7%) tested animals produced a positive reaction. The highest number of reactors (tuberculin-positive animals) and the highest percentage of isolation from truly infected animals were recorded in El-Sharkia Governorate (5.1%), followed by El-Qalyubia Governorate (4.2%) and Cairo (2.5%).

**Table 1:** Prevalence of bovine tuberculosis in dairy cows using single tuberculin tests at three different governorates.

Source	Number of tested cows	Positive tuberculin reactors		Negative tuberculin reactors	
	
		n	%	n	%
Cairo	1240	31	2.5	1209	97.5
El-Qalyubia	908	38	4.2	870	95.8
El-Sharkia	765	39	5.1	726	94.9
Total	2913	108		2805	

The intradermal tuberculin test is an inexpensive test used for TB diagnosis in cattle for both latent and active infections [5]. However, the method has limited sensitivity and specificity and is influenced by many factors related to immunological response [15]. Tuberculin potency varies between batches and significantly affects the number of revealed reactors [16]. Here, we report a higher prevalence of TB, perhaps due to limited implementation of biosecurity control and poor hygiene at the examined farms, where there was no clear plan to dispose of wastes and carcasses. The findings are also attributable to the management system, animal density, herd size, breeding, and differences in the geographical location that influence disease epidemiology [17].

**Figure-1:**
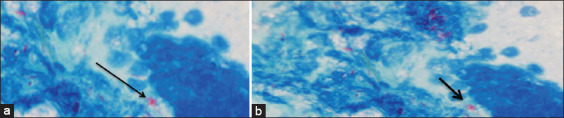
(a and b) Direct smear of lung from tuberculin reactor cow stained by Ziehl–Neelsen stain.

The conventional culture technique includes plating prepared samples on L-J and Middlebrook agar media. Among 108 tuberculin-positive SICTT reactors, 56 lymph node samples harbored mycobacterium isolates (51.85%). Of the 2805 (negative tuberculin) raw milk samples, 52 *Mycobacterium* spp. isolates were recovered (1.85%). Microscopic examination was conducted for all isolated strains using the modified Z-N stain (Table-2 and Figure-1). The results of the conventional culture technique were similar to results reported by Ofukwu *et al*. [18], with a percentage of 1.4%. Another report on market milk found one positive sample among 50 examined samples (2%) by culture [19]. Others have reported higher infection rates, ranging from 4.35% to 18.7% [20-22]. In this study, microscopic examination yielded results similar to those reported elsewhere [4], with rates of 2.56% in raw cow’s milk and 51.59% in lymph nodes. Microscopic examination of lymph nodes collected from tuberculin-positive animals by Z-N stain revealed *M. bovis* in 52/108 samples (48.1%), higher than the prior report, citing 21.43% [23]. The results are inconsistent with a report citing a 69% positivity rate [24]. Although the conventional *M. bovis* culture technique is still considered the gold standard in diagnosing bovine TB, it has lower sensitivity than other methods and is time-consuming, requiring a culture of up to 12 weeks [25]. Besides the frequency of false-negative results, the method requires extensive precautions to prevent contamination by related organisms [26]. Microscopic examination of clinical specimens is a simple, inexpensive, and relatively quick screening test for TB diagnosis [27]. Still, it also has many drawbacks, including inadequate sensitivity and contaminant overgrowth by related organisms during delayed sample transport [28]. Reliable detection requires a uniformly distributed bacterial load greater than 104 bacilli per milliliter [28].

**Table 2:** Detection of tuberculosis by conventional methods (cultivation findings and microscopical findings).

Source	Number of samples	Bacteriological findings			

		Cultivation finding		Microscopical findings	
	
		Number of isolates	%	Number of isolates	%
Lymph nodes	108	56	51.85	52	48.1
Raw milk	2805	52	1.85	30	1.06
Total	2913	108		82	

The advancement of practical serological techniques for diagnosing TB constitutes one of the most important problems in the veterinary medical profession [29]. Antibody-based diagnostic methods such as ELISA are directed toward the humoral immune response, characterized by antibody production. Otherwise, the cell-mediated immune response to TB pathogenesis involves IFN-γ and other cytokines [30]. Antibodies are only produced during the advanced phases of TB and usually cannot be detected in the early stages of the disease [31]. Single antigen testing also provides low detection sensitivity [6]. However, ELISA can be used to monitor the progress of infection [32] and can be used to confirm the results of skin tests [33]. It has been practically applied as a sensitive method for measuring antibodies in the sera of positive animals. The ELISA technique nevertheless yields variable sensitivity and specificity when matched to conventional culture methods and depending on the antigens used. In this study, ELISA using PPD antigen showed that 94 serum samples were positive (87.03%), whereas commercial polypeptide antigen showed that 97 serum samples were positive (89.81%) (Table-3). These results were similar to a prior report in Egypt that showed 90.6% positivity among tuberculin-positive reactors [34]. Another study in Egypt showed 14% positive serum samples out of 50 specimens examined using ELISA [19].

**Table 3:** Serodiagnosis of bovine tuberculosis by ELISA technique using bovine PPD and commercial polypeptide antigen comparison to the results of tuberculin test.

Source	Number of tuberculin- positive cows	ELISA			

		Polypeptide Ag.		Bovine PPD	
	
		+^ve^	%	+^ve^	%
Cairo	31	28	90.32	29	93.54
El-Qalyubia	38	34	89.47	35	92.10
El-Sharkia	39	32	82.05	33	84.61
Total	108	94		97	

ELISA=Enzyme-linked immunosorbent assay, PPD=Purified protein derivative

These results indicate that ELISA using commercial polypeptide antigen is more sensitive than ELISA using the traditional PPD antigen for bovine TB diagnostics. A cocktail of precisely selected antigens may be promising as a novel diagnostic reagent.

The IFN-γ assay is an *in vitro* technique that relies on cell-mediated immune activity. It exposes IFN-γ in PPD challenged whole blood cultures. The test scales the release of IFN-γ from sensitized blood lymphocytes stimulated by mycobacterial antigens using sandwich ELISA [35]. The assay only yields a positive result when a sample contains a detectable level of IFN-γ above the known and where there is a demonstration of complex *M. tuberculosis* cell-mediated immune responses. The IFN-γ test has several advantages over intradermal methods. It has better sensitivity (where standard SICTT is applied), with an increased ability to detect bovine TB than the intradermal skin test [36], and provides earlier detection than SICTT by 60-120 days.

Furthermore, it can be immediately repeated on the same animal, which can only be handled once [7]. The IFN-γ test is also directed to specific antigens absent in Bacillus Calmette-Guerin or other non-tuberculous mycobacteria [37]. The sensitivity rate of the IFN-γ assays based on PPD has been estimated to be 73.0-100% [36]. Use of bovis-specific antigens instead of PPD can increase the sensitivity of IFN-γ assay without decreasing its specificity [38].

**Figure-2:**
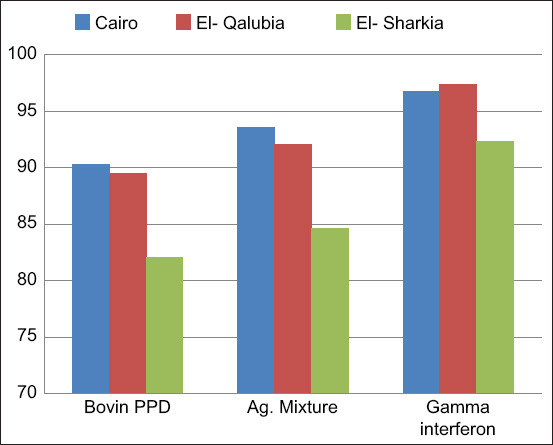
Comparison between enzyme-linked immunosorbent assay technique and gamma-interferon assay in tuberculin-positive cows at different governorates.

The IFN-γ results for 108 animals (whole blood samples) revealed that 103 (95.37%) were positive, including 36/39 (92.30%) in El-Sharkia Governorate, 37/38 (97.36%) in El-Qalyubia Governorate, and 30/31 (96.77%) in Cairo Governorate (Table-4). Figure-2 shows the comparison of the IFN-γ and ELISA results. We found that 94/108 (87.03%) were positive by ELISA using PPD antigen, 97/108 (89.81%) were positive by ELISA using commercial polypeptide antigen, and 103/108 (95.37%) were positive by IFN-γ assay. These results were consistent with a prior report showing that the IFN-γ assay was more sensitive in the diagnosis of *M. bovis* in blood-positive cows (83.3%) than Middlebrook 7H9 agar (55.5%) and ELISA using PPD-B antigen (72.2%) [39]. The performance of both IFN-γ tests and ELISA in a parallel survey facilitates the detection of a greater number of infected animals before they become a risk for other animals as a source of infection and environmental contamination [27]. The IFN-γ assay could disclose more positive cases, indicating that it is more sensitive than ELISA. In general, test sensitivity relies on the phase of infection in the study subject, so integrating tests can usually be advantageous in diagnosing bovine TB better [40]. Other studies have concluded that the IFN-γ assay may be useful for diagnosing TB [41].

**Table 4:** Results of gamma-interferon test on tuberculin-positive cows.

Governorates	Number of tuberculin-positive cows	Gamma -interferon	

		+^ve^	%
Cairo	31	30	96.77
El-Qalyubia	38	37	97.36
El-Sharkia	39	36	92.30
Total	108	103	

## Conclusion

The use of polypeptide antigen in ELISA provides a more efficient diagnosis than PPD-B antigen**.** The IFN-γ assay detected more positive cases than other tests, indicating that it is more sensitive than both SICTT and ELISA and should be used parallel to SICTT to detect more positive animals before they become a source of infection risk for other animals and humans. We conclude that no single test can detect all positive cases; hence, at least two tests are required to reach the highest possible specificity and sensitivity.

## Authors’ Contributions

SHAH: Conception and design of the study, laboratory work, and data analysis. AND and HMS: Supervised the study, Prepared and revised the manuscript. MBA: Collection of the samples and the laboratory work. FAK: Serological test. All authors read and approved the final manuscript.
